# Multiplex PCR-based epidemiology of viral acute respiratory infections in hospitalized children: implications for antibiotic stewardship in Saudi Arabia

**DOI:** 10.3389/fped.2026.1857382

**Published:** 2026-06-17

**Authors:** Yasser Sedky, Mohamed Abdulhay, Nouran sedky, Heba Omair, Shaimaa Abdalaleem Abdalgeleel, Sarah A. Saleh

**Affiliations:** 1 Department of Pediatrics, Mouwasat Hospital Khobar, Khobar, Saudi Arabia; 2 Department of Pediatrics, Cairo University, Cairo, Egypt; 3 Department of Pediatrics, Faculty of Medicine, Capital University (formerly Helwan University), Cairo, Egypt; 4 Department of Medical Molecular Genetics, Division of Human Genetics and Genome Research, National Research Centre, Cairo, Egypt; 5 Department of Clinical and Chemical Pathology, Faculty of Medicine, Cairo University, Cairo, Egypt; 6 Department of Public Health, Faculty of Applied Medical Sciences, Al-Baha University, Al-Baha, Saudi Arabia; 7 Department of Epidemiology and Biostatistics, National Cancer Institute, Cairo University, Cairo, Egypt

**Keywords:** acute respiratory infection, antimicrobial stewardship, multiplex PCR, paediatric epidemiology, RSV, Saudi arabia

## Abstract

**Background:**

Acute respiratory infections (ARIs) remain a leading cause of paediatric hospitalization worldwide. However, data describing viral epidemiology and management patterns in the Middle East remain limited.

**Objectives:**

To describe the epidemiology, clinical characteristics, monthly viral distribution, treatment practices, and antibiotic utilization patterns among hospitalized children with acute viral respiratory illness during the peak respiratory virus period.

**Methods:**

This retrospective observational study included 421 children younger than 14 years admitted to a tertiary care hospital in Saudi Arabia with febrile respiratory illness between October 2024 and February 2025. Demographic, clinical, laboratory, treatment, and multiplex respiratory PCR results were analysed. Statistical significance was set at *p* ≤ 0.05.

**Results:**

The mean age was 3.2 ± 2.9 years, and 58.2% were male. Overall, 42.8% required supplemental oxygen and 12.1% required ICU admission. Respiratory pathogens were detected in 72.9% of patients. The most common viruses were respiratory syncytial virus (RSV) (35.5%) and Enterovirus (22.1%). Viral positivity was significantly associated with younger age, longer hospital stays, and increased oxygen requirement. Inflammatory markers (CRP and ESR) and antibiotic use were significantly higher in patients with negative swab results (*p* < 0.05). RSV showed a marked seasonal peak in November, while Enterovirus circulated throughout the study period.

**Conclusion:**

RSV and Enterovirus were the predominant causes of paediatric ARI hospitalization during the winter in Saudi Arabia. Early viral identification using multiplex PCR may reduce unnecessary antibiotic use and improve antimicrobial stewardship. these findings support continued regional viral surveillance to guide seasonal preparedness and clinical management

## 1 Introduction

Acute respiratory infections (ARIs) are among the most common causes of illness and death in children worldwide and represent one of the leading causes of outpatient visits and hospital admissions globally (1–3). Viral pathogens, which account for up to 80% of pediatric ARIs, include respiratory syncytial virus (RSV), influenza viruses, parainfluenza viruses, rhinoviruses, and enteroviruses, represent their major role in disease burden (1–6). ARIs encompass a broad spectrum of illnesses, ranging from mild upper respiratory tract infections to severe lower respiratory tract infections (6–10). Influenza viruses exhibit predictable seasonal patterns, with higher incidence during winter (3, 11). Other viruses, including human metapneumovirus (hMPV), bocavirus, and parainfluenza viruses, demonstrate variable circulation patterns across different years (2, 10, 12, 13). RSV remains the leading cause of hospitalization among children under five years of age, particularly during cooler months (1, 5, 14). Despite limited data on pediatric ARI viral epidemiology in Saudi Arabia, regional insights are vital for pediatricians and epidemiologists to tailor effective interventions, guiding clinical practice and public health service (15, 16).

Real-time multiplex polymerase chain reaction (PCR) has become a cornerstone in the diagnosis of pediatric acute respiratory infections due to their high sensitivity and specificity, in addition to its ability to simultaneously detect multiple respiratory pathogens from a single clinical sample. Although rapid antigen detection tests (RADTs) provide shorter turnaround times as point-of-care tests, multiplex PCR assays offer substantially greater diagnostic accuracy, particularly in specimens with low viral loads where antigen-based tests may yield false-negative results (17). Recent studies have demonstrated higher detection rates of PCR-based assays compared with rapid antigen tests for respiratory viruses, including influenza, RSV, and SARS-CoV-2 infections (18).

The broad pathogen coverage and enhanced sensitivity of multiplex PCR facilitate precise identification of viral etiologies with overlapping clinical presentations and support appropriate clinical management and infection control strategies (19, 20).

Furthermore, accurate differentiation between viral and bacterial respiratory infections supports clinical decision-making, helps reduce unnecessary antibiotic prescriptions, and promotes antimicrobial stewardship in hospitalized children (21–24).

The expanded adoption of PCR-based respiratory panels in the post-pandemic era emphasizes its value in understanding shifting viral epidemiology, improving infection control, and guiding targeted therapeutic and management strategies in pediatric populations (13, 19–22).

This study aimed to retrospectively evaluate the clinical presentation, laboratory findings, viral etiology, and management of children hospitalized with ARIs during the winter season at a tertiary care center in Saudi Arabia.

## 2 Methods

### 2.1 Study design and setting

This retrospective observational study was conducted at Mouwasat Hospital in Al Khobar, Saudi Arabia, a tertiary care center. The study period extended from October 2024 to February 2025, corresponding to the peak winter respiratory infection season.

### 2.2 Study population

The study included hospitalized children aged 14 years or younger presenting with febrile respiratory illness. Acute respiratory infection (ARI) was defined as the presence of cough and/or respiratory distress, with or without fever.

### 2.3 Inclusion criteria

The following criteria were required for patient enrollment.

1. Age younger than 14 years.
2. Admitted to the pediatric inpatient ward during the study period.
3. Presented with fever and respiratory symptoms suggestive of acute respiratory infection (ARI), such as cough or respiratory distress.
4. Had clinical and/or laboratory findings consistent with acute respiratory infection, including respiratory symptoms, supportive inflammatory markers, radiologic findings when available, and/or positive respiratory multiplex PCR testing.
5. Sufficient clinical data was available for severity score assessment. The Paediatric Respiratory Severity Score (PRESS) was used as a scoring system. (PRESS) was selected because it is a validated, practical bedside tool for assessing respiratory illness severity in children and is suitable for retrospective clinical data analysis

During the study period, the study investigators did not enroll patients according to a predefined interventional research protocol. Rather, this was a retrospective observational analysis of hospitalized children who underwent clinically indicated multiplex respiratory PCR testing as part of standard hospital care during the winter respiratory virus season.

### 2.4 Exclusion criteria

Patients were excluded if they met any of the following criteria:

1. Admission was primarily due to a non-respiratory condition (e.g., surgical, gastrointestinal, or neurological disease), even if respiratory-like symptoms were present at initial evaluation. Some of these patients underwent multiplex PCR testing during the initial workup but were excluded from the final analysis after acute respiratory infection was ruled out.
2. No clinical or laboratory evidence of acute respiratory infection was present.
3. Medical records were incomplete or missing essential study data.

### 2.5 Data collection and variables

Demographic, clinical, laboratory, and microbiological data were extracted from electronic medical records. Collected demographic variables included age, sex, nationality, weight, height, and length of hospital stay.

Clinical variables included presenting symptoms, degree of respiratory distress, oxygen requirement, ICU admission, body temperature, oxygen saturation, and disease severity assessment using (PRESS) Score. Respiratory distress was defined clinically by the presence of tachypnea, chest retractions, nasal flaring, grunting, increased breathing effort, or hypoxemia requiring supplemental oxygen, as documented in the medical record.

Laboratory parameters included total leukocyte count, hemoglobin level, platelet count, erythrocyte sedimentation rate (ESR), C-reactive protein (CRP), and serum electrolytes (sodium and potassium). Given the overall volume of pediatric admissions (821 of 6,118 pediatric ER visits), PCR testing represents a relatively small proportion of total care costs. It may support more targeted management and improved resource utilization.

Treatment-related variables included antibiotic use, corticosteroid therapy, nebulization therapy, and respiratory support measures when required.

### 2.6 Sample collection and handling

Nasopharyngeal swabs were collected upon admission as part of routine clinical care using sterile swabs and placed in viral transport medium (VTM). Samples were transported under standard biosafety conditions and processed promptly upon receipt.

### 2.7 Multiplex real-time PCR testing

Respiratory samples were analyzed using the QIAGEN QIAstat-Dx Respiratory Panel on the QIAstat-Dx Analyzer platform. This multiplex real-time RT-PCR assay targets conserved genomic regions specific to each pathogen included in the panel. The targeted regions include viral genes encoding structural or replication-associated proteins, such as nucleocapsid, matrix, envelope, and polymerase genes, as well as bacterial species-specific genes. Representative gene targets included influenza matrix genes, RSV nucleoprotein genes, SARS-CoV-2 envelope and RNA-dependent RNA polymerase genes, and species-specific bacterial genomic sequences. The assay enables simultaneous qualitative detection of a broad spectrum of respiratory pathogens.

Sample preparation, nucleic acid extraction, reverse transcription, amplification, and result interpretation were performed automatically by the integrated analyzer system according to the manufacturer's instructions. Internal procedural controls incorporated within each cartridge were used to verify specimen adequacy, extraction efficiency, and amplification validity. A test result was considered positive when a valid amplification curve with a cycle threshold (Ct) value within the manufacturer-defined acceptable range was detected for the corresponding pathogen target. Invalid results were repeated in accordance with laboratory quality control procedures.

The QIAstat-Dx Respiratory Panel has demonstrated high analytical performance in manufacturer validation studies and published evaluations, with reported sensitivity and specificity generally exceeding 95% for most viral and bacterial respiratory targets.

### 2.8 Testing strategy and cost context

During the study period, PCR testing for respiratory pathogens was part of the routine diagnostic protocol for all pediatric patients admitted with clinically suspected ARI, regardless of severity, to support infection prevention measures and guide antimicrobial decisions.

The average institutional cost of one QIAstat-Dx multiplex respiratory panel test during the study period was approximately 450 Saudi Riyals (≈120 USD) per cartridge, including reagents, consumables, and instrument use. Although this represents a notable per-test cost, it was offset by its contribution to improved diagnostic accuracy, reduced isolation time, and potentially more rational antibiotic use.

### 2.9 Result interpretation criteria of positivity

**Figure 1:**
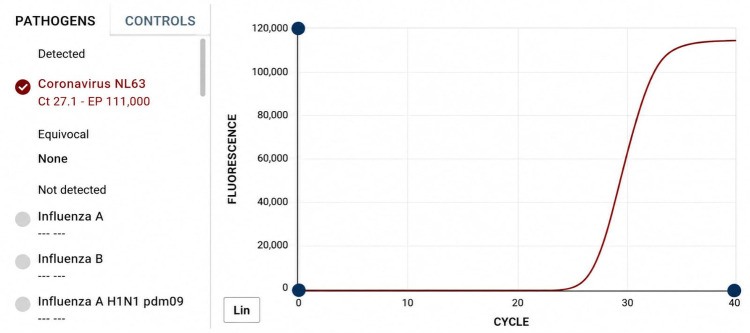
Representative amplification plot of coronavirus NL63 detected by the QIAstat-Dx respiratory panel showing a positive RT-PCR signal with a Ct value of 27.1.

A result for a respiratory organism is interpreted as “Positive” when the corresponding PCR assay is positive, except for Influenza A. The Influenza A assay in the QIAstat-Dx Respiratory SARS-CoV-2 Panel is designed to detect Influenza A as well as Influenza A subtype H1N1/2009, Influenza A subtype H1, or Influenza A subtype H3. For every other pathogen that can be detected with the QIA stat-Dx Respiratory SARS-CoV-2 Panel, only one signal will be generated if the pathogen is present in the sample Figure 1.

### 2.10 Sensitivity and specificity

According to the manufacturer, the QIAstat Dx Respiratory Panel demonstrated an overall sensitivity of 97.2% and specificity of 96.1%, with high agreement across most individual “pathogens. Additionally,” assay performance may vary with pathogen prevalence.

### 2.11 Outcomes

The primary outcome was the prevalence and distribution of respiratory pathogens among hospitalized children with ARIs. Secondary outcomes included associations between PCR positivity and demographic characteristics, clinical presentation, laboratory findings, and treatment patterns. Also included patterns of antibiotic utilization in relation to PCR results and their potential implications for antimicrobial stewardship.

### 2.12 Statistical analysis

Data were analyzed using SPSS version 28. Categorical variables were expressed as frequencies and percentages, while continuous variables were presented as mean ± standard deviation. The chi-square test was used to assess associations between categorical variables, and the Mann–Whitney *U*-test was used to compare continuous variables between groups. Logistic regression was used to estimate odds ratios, and 95% confidence intervals were calculated for factors associated with a positive swab result. A *p*-value ≤ 0.05 was considered statistically significant.

## 3 Results

During the study period (October 2024 to February 2025), all included patients represented hospitalized pediatric ARI cases who underwent PCR testing based on clinical indication. The final analyzed cohort consisted of 421 hospitalized children with complete clinical and laboratory records available for retrospective review.

### 3.1 Sociodemographic and clinical characteristics

A total of 421 participants were included in the study. The mean age was 3.2 ± 2.9 years, with a median of 2 years (range: 0.3–13). Males represented 58.2% (*n* = 245), while females accounted for 41.8% (*n* = 176). Most participants were Saudi nationals (64.6%), and 35.4% were non-Saudi. The mean length of hospital stay was 3.6 ± 1.9 days (median: 3; range: 1–30). Weight-for-age and height-for-age Z-scores were both approximately normal (0.0 ± 0.9). Regarding disease severity stratification using PRESS scoring, most patients were classified as score 2 (56.3%), followed by score 3 (23.3%), score 5 (11.4%), and score 4 (9.0%).

PCR testing was routinely performed for hospitalized pediatric patients with clinically suspected ARI according to institutional protocol during the study period, particularly in hospitalized children, where results may influence management, infection control, or antibiotic stewardship.

Regarding healthcare utilization, 12.1% of patients required ICU admission, while 87.9% had mild-to-moderate disease. The estimated cost of a multiplex PCR test in our facility is approximately 120 USD, compared with an average hospitalization cost of 2,500–5,000 USD per admission. Given the overall volume of pediatric admissions (821 of 6,118 pediatric ER visits), PCR testing represents a relatively small proportion of total care costs. It may support more targeted management and improved resource utilization (Table 1).

**Table 1:** Sociodemographic characteristics of the participants (*n* = 421).

Characteristics	Total (*n* = 421)
Age (years)	
Mean ± SD	3.2 ± 2.9
Median (range)	2 (0.3–13)
Gender	
Female	176 (41.8%)
Male	245 (58.2%)
Nationality	
Saudi	272 (64.6%)
-Saudi	149 (35.4%)
Length of hospital stay (day)	
Mean ± SD	3.6 ± 1.9
Median (range)	3 (1–30)
Z score for weight	
Mean ± SD	.0 ± 0.9
(range)	−0.2 (−3.3–4.9)
Z score for height	
Mean ± SD	0.0 ± 0.9
Median (range)	0.1 (−4.1–2.8)
PRESS scoring	
2	237 (56.3%)
3	98 (23.3%)
4	38 (9%)
5	48 (11.4%)

### 3.2 Clinical presentation and laboratory findings

All participants (100%) presented with cough. Oxygen supplementation was required in 42.8% of cases, while 19.7% developed respiratory distress, and 12.1% required ICU admission. The mean temperature was 37.1 ± 0.7 °C, and oxygen saturation averaged 97.5 ± 2.0%. Laboratory findings showed a mean total leukocyte count of 4.1 ± 3.1, hemoglobin 11.7 ± 1.1 g/dL, platelet count 336.1 ± 126.3, ESR 26.5 ± 21.3, and CRP 23.9 ± 40.1. Electrolytes were within normal ranges (sodium: 139.9 ± 2.3; potassium: 4.5 ± 0.7). Supplementary Table S1.

### 3.3 Respiratory pathogen detection (swab results)

**Figure 2:**
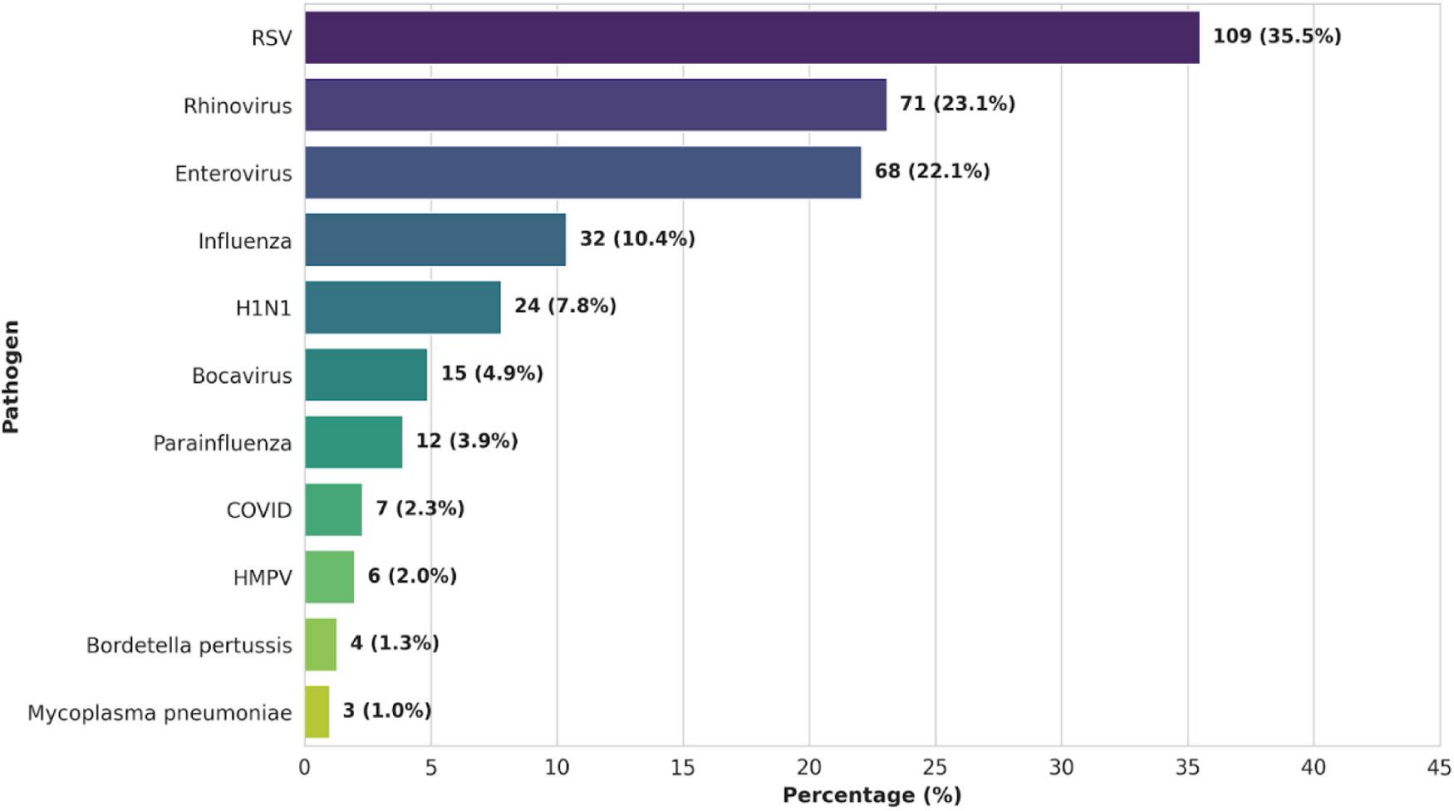
Shows the results of positive swabs regarding organisms.

Out of 421 samples, 72.9% (*n* = 307) were positive, while 27.1% (*n* = 114) were negative. Multiple pathogens were identified among positive “cases. Respiratory” viral infections were highly prevalent among the studied children, with 72.9% of swabs testing positive. Among the detected pathogens, Respiratory syncytial virus was the most frequently identified organism, accounting for 35.5% of positive swabs, followed by Rhinovirus (23.1%) and Enterovirus (22.1%). Influenza A (H1N1) and other monthly viral pathogens were also commonly detected, highlighting the substantial contribution of monthly viral pathogens to acute respiratory infections during the study period. Less frequently identified organisms included Parainfluenza, COVID-19, Human metapneumovirus infection, Bordetella pertussis infection, and Mycoplasma pneumonia. The findings also indicate the presence of co-infections, as positive swabs could contain more than one detected organism (Table 2, Figure 2).

**Table 2:** Swab results with pathogen distribution.

Characteristics	**Total (*n*** **=** **421)**
Swab results	
Negative	114 (27.1%)
Positive	307 (72.9%)
Results of positive swab (*n* = 307) may have more than one organism.	
Mycoplasma pneumoniae	3 (1%)
Bordetella pertussis	4 (1.3%)
HMPV	6 (2%)
COVID	7 (2.3%)
Parainfluenza	12 (3.9%)
Bocavirus	15 (4.9%)
H1N1	24 (7.8%)
Influenza	32 (10.4%)
Enterovirus	68 (22.1%)
Rhinovirus	71 (23.1%)
RSV	109 (35.5%)

Co-detection with bacterial pathogen was detected in few cases including Bordetella pertussis and Mycoplasma pneumoniae (1.3%), (1%), and other bacterial pathogens were not fully presented but indicated through elevated inflammatory markers. Antibiotic use showed a statistically significant association with the swab results (*p* = 0.040). A higher proportion of patients in the negative group received antibiotics than in the positive group (74.1% vs. 63.4%), whereas patients who did not receive antibiotics were more frequent in the positive group (36.6% vs. 25.9%).

### 3.4 Monthly distribution of swab positivity

**Figure 3:**
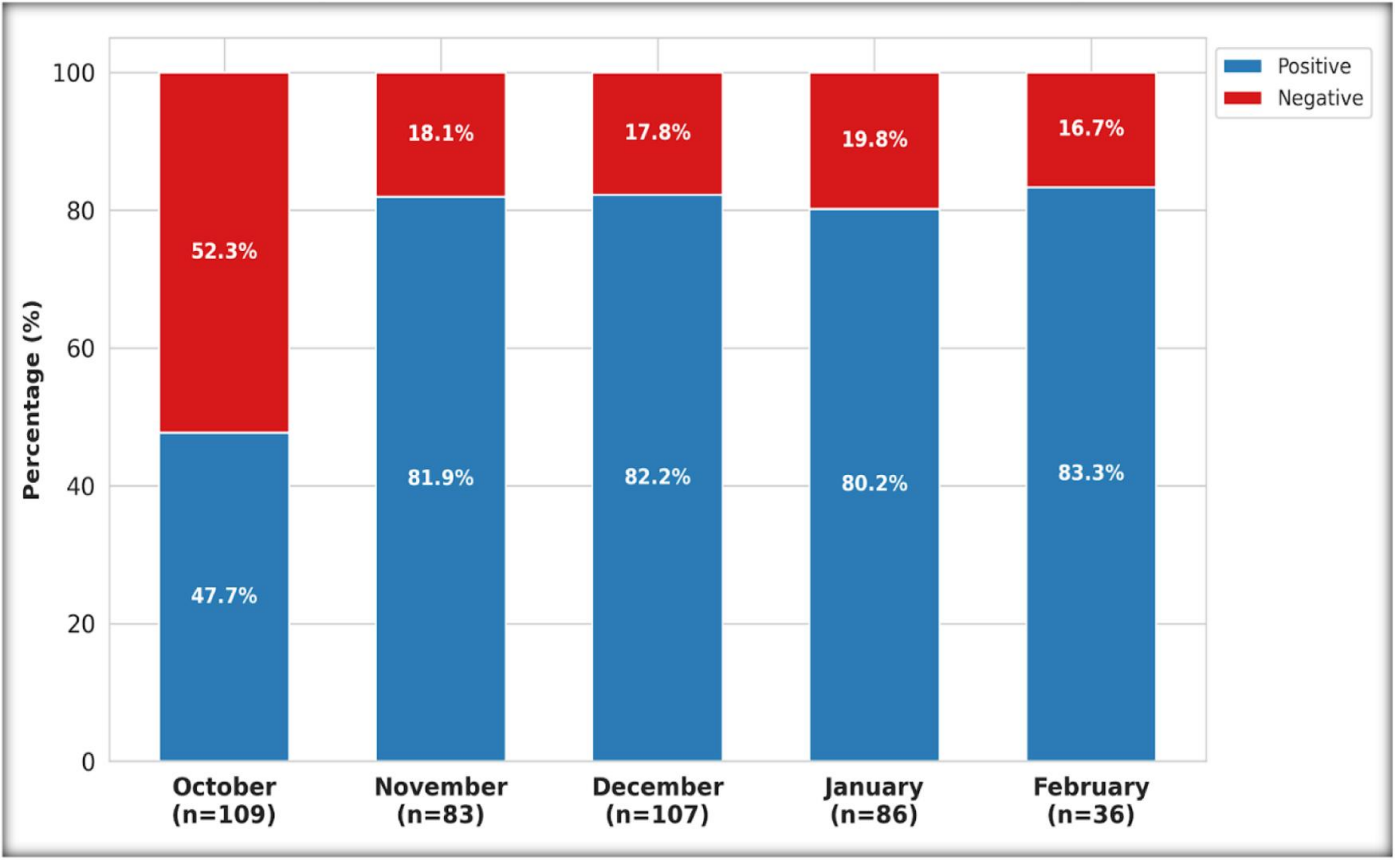
Monthly swab distribution.

There was a clear monthly distribution in respiratory pathogen distribution across the study months, with a consistently high proportion of positive swabs ranging from 47.7% in October to 83.3% in February. RSV, rhinovirus, and enterovirus were the most frequently detected pathogens throughout the study period, with RSV showing the highest overall prevalence, particularly in November (42.6%) and January (43.4%). Rhinovirus also remained consistently common throughout all months, peaking in January (29.0%), while enterovirus showed relatively stable detection with higher proportions in December (29.5%) and February (26.7%). Influenza viruses, including H1N1 and seasonal influenza, demonstrated marked variability, with higher detection in October and December, suggesting early monthly circulation. COVID-19 was detected only in October, while HMPV was identified exclusively in November. Less frequent pathogens such as Mycoplasma pneumoniae and Bordetella pertussis were observed only in February. Overall, the findings highlight the predominance of RSV and other common respiratory viruses with distinct monthly fluctuations and the presence of multiple co-circulating pathogens during the study period. Supplementary Table S2, Figure 3.

### 3.5 Comparison between positive and negative swab groups (sociodemographic factors)

Patients with positive swabs were significantly younger than those with negative results (2.9 ± 2.8 vs. 3.9 ± 3.4 years, *p* = 0.002). A slightly longer hospital stay was observed in the positive group (3.6 ± 1.5 vs. 3.4 ± 2.9 days, *p* = 0.007). No statistically significant differences were observed in gender, nationality, Z-scores for weight-for-age or height-for-age between the groups (*p* > 0.05). or PRESS score (*p* > 0.05). Supplementary Table S3.

### 3.6 Clinical and laboratory comparison by swab status

Patients with positive swabs required oxygen therapy more frequently (46.3% vs. 33.3%, *p* = 0.017).

Inflammatory markers were significantly lower in the positive group, including ESR (23.3 ± 18.5 vs. 32.8 ± 25.1, *p* = 0.035) and CRP (20.1 ± 33.0 vs. 34.3 ± 53.7, *p* = 0.009). No significant differences were observed in temperature, oxygen saturation, respiratory distress, ICU admission, leukocyte count, hemoglobin, platelet count, sodium, or potassium levels (*p* > 0.05) Supplementary Table S4.

### 3.7 Diagnostic categories and treatment protocols

The study cohort encompassed a spectrum of acute respiratory infections, including bronchiolitis (41.6%), viral pneumonia (28.3%), wheezing-associated illnesses (17.6%), laryngotracheitis or croup (7.8%), and acute upper respiratory tract infections (4.7%).

In our institution, during the study period, nebulized therapy was routinely prescribed for children with cough and wheezing as part of standard supportive management of bronchiolitis and viral lower respiratory tract infections. The nebulized agents included normal saline (approximately 70% of cases), salbutamol (20%), and adrenaline (10%), the latter mainly in patients with croup or stridor. The widespread use of nebulization reflects historical clinical practice and institutional preference rather than individualized bronchodilator indication. Corticosteroids were administered in 45.6% of patients, predominantly in those with croup, asthma-like wheezing, or a history of reactive airway disease. The main formulations were inhaled budesonide or systemic dexamethasone given as a single or short course, consistent with national treatment protocols for these conditions.

### 3.8 Multivariable logistic regression analysis

Multivariable analysis identified oxygen supply as a significant predictor of positive swab results (OR = 1.59, 95% CI: 1.4–2.5, *p* = 0.049). Age was inversely associated with positivity (OR = 0.92, 95% CI: 0.8–0.9, *p* = 0.026), indicating that younger patients were more likely to test positive. CRP was also negatively associated with swab positivity (OR = 0.99, 95% CI: 0.8–0.9, *p* = 0.008) (Table 3).

**Table 3:** Multivariable logistic regression analysis of factors associated with positive swab.

Predictors of positive swab results	*p*-value	OR	95% CI for OR	
			Lower	Upper
oxygen supply	.049	1.594	1.4	2.5
CRP	.008	.993	.8	.9
Age	.026	.922	.8	.9

### 3.9 Summary

These findings suggest that viral ARIs, predominantly caused by RSV and Enterovirus, are the leading etiology of pediatric hospitalizations during the winter period. Younger age and increasing oxygen requirements were associated with positive results. The elevated levels of inflammatory markers and increased antibiotic use in swab-negative patients suggest alternative infectious etiologies, underscoring the importance of appropriate viral diagnostics to guide management and of rational antibiotic use to reduce unnecessary exposure.

## 4 Discussion

Acute respiratory infections (ARIs) remain a leading cause of pediatric morbidity and hospitalization worldwide, particularly among children under five years of age. Viral pathogens continue to account for most severe lower respiratory tract infections and impose a substantial burden on healthcare systems despite advances in diagnostics and preventive strategies (1–3, 23). Large-scale epidemiological studies have consistently demonstrated the major contribution of RSV, influenza, and other respiratory viruses to pediatric hospitalizations and mortality, particularly in low- and middle-income countries (1–3, 18).

In the present study, viral pathogens were detected in 72.9% of hospitalized children with ARIs, confirming the strong predominance of viral etiologies. This is consistent with multicenter and retrospective studies using multiplex PCR platforms, which have shown improved pathogen detection rates compared with conventional diagnostic methods (8, 17, 19, 21, 22). The growing use of molecular diagnostics has enhanced respiratory pathogen detection in pediatric infections (19, 20).

RSV was the most frequently identified pathogen (35.5%), followed by Enterovirus (22.1%) and influenza A (H1N1) (7.8%). RSV is well established as the leading cause of severe lower respiratory tract infections in infants and young children globally (1, 5, 14, 25). Its clinical severity, including bronchiolitis and hypoxemic respiratory failure, has been consistently reported in both high-income and resource-limited settings (5, 10, 14, 25, 26). In our cohort, this is reflected by the high proportion of oxygen-requiring patients.

Influenza virus remains an important pathogen contributing to pediatric hospitalizations worldwide (3, 9). Comparative studies have demonstrated that RSV generally causes more severe disease than influenza or parainfluenza viruses in early childhood (22, 27–29). Parainfluenza viruses also contribute significantly to pediatric respiratory morbidity, with variable severity depending on viral subtype (2, 7, 29).

Enteroviruses accounted for a substantial proportion of infections in this study. Recent surveillance data indicate an increasing role of Enteroviruses and rhinoviruses in pediatric respiratory infections, particularly in post-pandemic periods (8, 15, 19, 28). This rise may reflect changes in viral circulation patterns following COVID-19-related disruptions and immunity gaps (12, 17, 18). Unlike RSV, Enterovirus demonstrates less pronounced seasonality and circulates year-round (4, 21), which explains its consistent detection across study months.

Other pathogens detected included human metapneumovirus (HMPV), bocavirus, Mycoplasma pneumoniae, and Bordetella pertussis. HMPV is a recognized cause of pediatric respiratory disease with clinical features like RSV (11). Bocavirus is frequently co-detected and may not always be a primary pathogen (28). Mycoplasma pneumoniae and Bordetella pertussis remain less common but clinically important causes of pediatric respiratory infections (14, 27). Viral co-circulation patterns observed in this study are consistent with global pediatric epidemiological data (14, 27).

Younger age was significantly associated with viral positivity, with infected children being younger than those in the negative group. This aligns with established evidence showing increased susceptibility in infants and toddlers due to immature immunity and smaller airway diameter (1, 3, 23). (PRESS) score was applied to assess disease severity (30). Similar findings have been reported across multiple multicenter studies (14, 27, 30).

Cough was a universal symptom among all patients, and 42.8% needed oxygen support, highlighting a significant burden of moderate-to-severe respiratory issues. The need for oxygen was independently associated with viral positivity, underscoring the clinical importance of RSV and enterovirus in hypoxemic respiratory illness (5, 21, 25).

Inflammatory markers showed an inverse association with viral infection, with CRP and ESR significantly higher in swab-negative patients. This highlights the diagnostic challenge of differentiating viral from bacterial infections using routine laboratory markers alone, which often leads to empirical antibiotic use (22).

An important finding was that 63.4% of children with confirmed viral infection still received antibiotics. This reflects real-world clinical practice in hospitalized pediatric patients, where initial antibiotic therapy is frequently started empirically at admission due to the difficulty of excluding bacterial co-detection at early presentations. Importantly, PCR testing in our study was not implemented within a prospective antimicrobial stewardship intervention but was analyzed retrospectively as part of routine clinical care. Therefore, the observed antibiotic prescribing patterns likely represent baseline institutional practice prior to structured stewardship implementation. This supports the need for continued surveillance to monitor shifts in pathogen prevalence and healthcare preparedness.in addition it helped us to limit the duration of unnecessary antibiotic use.

RSV showed a clear monthly peak in November and December, consistent with global RSV monthly circulation pattern (4, 13). Influenza demonstrated broader monthly distribution pattern (3, 9), while Enterovirus showed prolonged activity across multiple months, consistent with its known epidemiological behavior (19, 28).

Post-pandemic changes in viral circulation have been widely documented, including altered seasonality and resurgence of respiratory viruses following relaxation of public health measures (12, 15, 16). These shifts likely contributed to the observed distribution of Enterovirus and other respiratory pathogens in this study.

Nebulizer therapy was the most frequently used treatment, reflecting standard supportive care for pediatric bronchiolitis and lower respiratory tract infections. Nebulized therapy mainly consisted of isotonic saline or salbutamol for bronchiolitis and wheezing management, according to national protocols. Corticosteroids, primarily dexamethasone or budesonide, were used in children with suspected croup or reactive airway disease (2, 18, 31).

Oxygen therapy was significantly more common in patients with viral positivity, consistent with RSV-associated disease severity (5, 24). However, ICU admission and respiratory distress did not differ significantly between groups, suggesting that multiple host and clinical factors influence progression to severe disease. The relatively high Enterovirus contribution compared with earlier regional reports may reflect an evolving post-pandemic respiratory viral landscape (12, 17, 18). This supports the need for continued surveillance to monitor shifts in pathogen prevalence and healthcare preparedness.

Rapid identification of viral pathogens may help reduce unnecessary antibiotic use and potentially support antimicrobial stewardship efforts (19–22). This is particularly important in pediatric populations, where distinguishing viral from bacterial infections remains challenging (22).

In addition, global modeling studies suggest that RSV vaccination strategies could significantly reduce pediatric respiratory disease burden in the future (32). Integration of vaccination and improved diagnostics may substantially change disease patterns in the coming years.

Although ARI have been extensively studied globally (1–3, 23, 24), this study provides updated regional evidence from a post-pandemic cohort. Similar epidemiological patterns have been observed in international studies describing variability in viral dominance and monthly distribution (6, 14, 27). Differences across regions underscore the importance of local surveillance to guide clinical practice and public health planning.

In Saudi Arabia, the National Immunization Program includes routine childhood vaccines such as BCG, hepatitis B, polio, DTP-Hib-HepB, pneumococcal conjugate vaccine (PCV), rotavirus, MMR, varicella, and meningococcal vaccines. At the same time, seasonal influenza vaccination is available and recommended for high-risk groups, but not universally mandated. Our study found a substantial burden of RSV-associated hospitalizations (35.5% of positive cases) in eastern Saudi Arabia. However, unlike recent European studies showing reduced RSV-related hospitalizations following implementation of preventive measures such as nirsevimab, no national RSV immunization program or routine preventive strategies were available in Saudi Arabia during the study period (October 2024–February 2025). Therefore, the high burden observed likely reflects baseline RSV morbidity in an unprotected population and may serve as a useful pre-intervention benchmark as Saudi Arabia considers introducing RSV preventive measures into the National Immunization Program (33).

Artificial intelligence is increasingly being explored in respiratory medicine as a supportive tool to improve diagnostic accuracy.

Emerging AI-based clinical decision-support systems may further enhance the interpretation of multiplex respiratory diagnostics and risk stratification in pediatric ARIs. However, current pediatric evidence remains preliminary (34).

### 4.1 Study limitations

This study has several limitations. Its retrospective design may introduce information bias and depend on the completeness and accuracy of medical documentation. As a single-center study, the findings may not fully reflect viral trends across other regions or healthcare institutions. Not all respiratory viral pathogens were systematically tested, potentially leading to an underestimation of the prevalence of common etiologies. The study did not comprehensively assess viral or bacterial co-detection, which may influence clinical severity. Restricting the cohort to hospitalized patients may also overrepresent severe ARI presentations. Finally, the absence of long-term post-discharge follow-up limits evaluation of clinical outcomes beyond hospitalization. Despite these limitations, the study provides valuable insights into viral epidemiology and clinical management patterns within this population. Future multicenter and outcome-focused studies are needed to address these important questions.

## 5 Conclusion

This study demonstrates the substantial burden of viral respiratory infections among hospitalized children in Saudi Arabia and supports the clinical value of PCR for improving diagnostic accuracy. Our findings highlight the need to revise local treatment protocols for pediatric acute respiratory infections and strengthen antimicrobial stewardship strategies. Education and reinforcement of evidence-based prescribing practices may reduce unnecessary antibiotic use and support future national surveillance initiatives. These data may also inform public health stakeholders and the National Immunization Technical Advisory Group regarding the potential value of RSV preventive strategies in Saudi Arabia.

### 5.1 Recommendations

Broader implementation of rapid multiplex viral testing PCR in hospitals to support pathogen-directed clinical decisions.

− Continued prioritization of preventive strategies, particularly seasonal influenza vaccination for children and RSV immunoprophylaxis for high-risk infants.
  Strengthening infection prevention and control measures in pediatric healthcare settings to limit in-hospital viral transmission.
− Enhancing clinician awareness and adherence to evidence-based ARI management guidelines, with emphasis on antibiotic and steroid stewardship.
− Maintaining continuous epidemiological surveillance to monitor evolving viral trends and inform monthly hospital preparedness planning.

## Data Availability

The original contributions presented in the study are included in the article/Supplementary Material, further inquiries can be directed to the corresponding author.
