# Supplementary material for: Multiplex PCR-based epidemiology of viral acute respiratory infections in hospitalized children: implications for antibiotic stewardship in Saudi Arabia
Source: Front Pediatr. 2026 Jun 17;14:1857382. doi: 10.3389/fped.2026.1857382 (PMC13318997; doi:10.3389/fped.2026.1857382)
Supplement: Supplementary file 1 [file Datasheet1.pdf]

Supplementary Table 1: Symptoms and signs, laboratory results among the participants

**Characteristics**	**Total (n=421)**
**Temperature**	
Mean ± SD	37.1±.7
**Oxygen**	
Mean ± SD	97.5±2.0
Median (range)	98 (80-100)
**Cough**	421 (100%)
**Oxygen supply**	180 (42.8%)
**Respiratory distress**	83 (19.7%)
**ICU admission**	51 (12.1%)
**Total leucocyte count**	
Mean ± SD	4.1±3.1
Median (range)	3.4 (.2-19)
**Haemoglobin**	
Mean ± SD	11.7±1.1
Median (range)	11.5 (4.2-14.7)
**Platelet**	
Mean ± SD	336.1±126.3
Median (range)	312 (14.2-925)
**ESR**	
Mean ± SD	26.5±21.3
Median (range)	20 (1-100)
**CRP**	
Mean ± SD	23.9±40.1
Median (range)	7.2 (.5-355)
**Sodium**	
Mean ± SD	139.9±2.3
Median (range)	140 (133-153)
**Potassium**	
Mean ± SD	4.5±.7

Median (range)	4.4 (2-10)

Supplementary Table2; Monthly distribution of swabs and pathogen identification.

Characteristics	October (n=109)	November (n=83)	December (n=107)	January (n=86)	February (n=36)
**Swab**					
Negative	57(52.3%)	15(18.1%)	19(17.8%)	17 (19.8%)	6 (16.7%)
Positive	52 (47.7%)	68 (81.9%)	88 (82.2%)	69 (80.2%)	30 (83.3%)
**Results of positive swab (may have more (than one organism), % from positive swab**					
Mycoplasma pneumonia	0 (0%)	0 (0%)	0 (0%)	0 (0%)	3 (10%)
Bordetella pertussis	0 (0%)	0 (0%)	0 (0%)	0 (0%)	4 (13.3%)
HMPV	0 (0%)	6 (8.8%)	0 (0%)	0 (0%)	0 (0%)
COVID	7 (13.5%)	0 (0%)	0 (0%)	0 (0%)	0 (0%)
Parainfluenza	2 (3.8%)	1 (1.5%)	2 (2.3%)	2 (2.9%)	5 (16.7%)
Bocavirus	1 (1.9%)	4 (5.9%)	3 (3.4%)	7 (10.1%)	0 (0%)
H1N1	7 (13.5%)	2 (2.9%)	12 (13.6%)	0 (0%)	3 (10%)

Influenza	9 (17.3%)	4 (5.9%)	10 (11.4%)	9 (13%)	0 (0%)
Enterovirus	14 (26.9%)	10 (14.7%)	26 (29.5%)	10 (14.5%)	8 (26.7%)
Rhinovirus	12 (23.1%)	18 (26.5%)	18 (20.4%)	20 (29.0%)	5 (16.7%)
RSV	15 (28.8%)	29 (42.6%)	25 (28.4%)	30 (43.4%)	10 (33.3%)

Supplementary Table3: Relation between sociodemographic and swab results

**Characteristics**	**Negative (n=114)**	**Positive (n=307)**	**p-value**
**Age (years)**			
Mean ± SD	3.9±3.4	2.9±2.8	**.002**
Median (range)	3 (.3-13)	2 (.3-13)	
**Gender**			
Female	45 (39.5%)	131 (42.7%)	.554
Male	69 (60.5%)	176 (57.3%)	
**Nationality**			.402
Saudi	70 (61.4%)	202 (65.8%)	
Non-Saudi	44 (38.6%)	105 (34.2%)	
**Length of hospital stay (day)**			
Mean ± SD	3.4±2.9	3.6±1.5	**.007**
Median (range)	3 (1-30)	3 (1-12)	
**Z score for weight**			
Mean ± SD	.1±.9	-.02±.9	
Median (range)	-.1 (-2.3-4.9)	-.2 (-3.3-3.7)	.207
**Z score for height**			

Mean ± SD	.04±1.02	-.01±.92	.199
Median (range)	.2 (-3.9-2.4)	.06 (-4.1-2.8)	
**PRESS scoring**			
2	72 (63.2%)	165 (53.7%)	.315
3	24 (21.1%)	74 (24.1%)	
4	9 (7.9%)	29 (9.4%)	
5	9 (7.9%)	39 (12.7%)	

Supplementary Table 4: Relationship between signs, laboratory results, and swab outcomes among participants, highlighting significant associations

	**Negative swab (n=114)**		**Positive swab (n=307)**		
**Characteristics**	**Mean ± SD**	**Median (range)**	**Mean ± SD**	**Median (range)**	**p-value**
**Temperature**	37.1±.7	36.9 (36-40.5)	37.4±.6	36.9(30.5-39.8)	.550
**Oxygen**	97.4±2.4	98 (80-100)	97.6±1.9	98 (91-100)	.492
**Oxygen supply n (%)**	38 (33.3%)		142 (46.3%)		**.017**
**Respiratory distress n (%)**	18 (15.8%)		65 (21.2%)		.217
**ICU admission n (%)**	10 (8.8%)		41 (13.4%)		.200
**Total leucocyte count**	4.1±3.1	3.3 (.3-18.4)	4.1±3.14	3.4 (.2-19)	.963
**Haemoglobin**	11.6±1.2	11.6 (7.8-14.3)	11.5±1.1	11.5 (4.2-14.7)	.930
**Platelet**	348.6±132.9	323.5 (54-925)	331.5±123.7	304 (14.2-807)	.209
**ESR**	32.8±25.1	25 (2-100)	23.3±18.5	17.5 (1-90)	**.035**
**CRP**	34.3±53.7	13.1 (.5-301.4)	20.1±33.0	13.1 (.5-301.4)	**.009**

**Sodium**	140±2.2	140 (135-146)	139.8±2.4	140 (133-153)	.613
**Potassium**	4.5±.8	4.4 (2.1-10)	4.4±.6	4.4 (2-6.7)	.701
